# Febuxostat, a Xanthine Oxidoreductase Inhibitor, Decreases NLRP3-dependent Inflammation in Macrophages by Activating the Purine Salvage Pathway and Restoring Cellular Bioenergetics

**DOI:** 10.1038/s41598-019-53965-x

**Published:** 2019-11-21

**Authors:** Johji Nomura, Tsunefumi Kobayashi, Alexander So, Nathalie Busso

**Affiliations:** 1 Pharmacology Research Department, Teijin Institute for Bio-medical Research, Teijin Pharma Limited, Hino, Tokyo Japan; 2 Service of Rheumatology, Department of Musculoskeletal Medicine, Centre Hospitalier Universitaire Vaudois, University of Lausanne, Lausanne, Switzerland

**Keywords:** Bioenergetics, Inflammasome

## Abstract

The nucleotide-binding oligomerization domain–like receptor family, pyrin domain–containing 3 (NLRP3) inflammasome mediates caspase-1 activation and IL-1β processing and is implicated in autoinflammatory as well as other chronic inflammatory diseases. Recent studies have demonstrated that xanthine oxidoreductase (XOR) inhibition attenuated IL-1β secretion in activated macrophages, but the detailed mechanism of inhibition remains unclear. In this study, we report that febuxostat, an inhibitor of XOR, suppressed NLRP3 inflammasome-mediated IL-1β secretion and cell death by two mechanisms: in a mitochondrial ROS (mitoROS)-dependent and mitoROS-independent manner. MitoROS-independent effects of febuxostat were mediated by an increase of intracellular ATP and improved mitochondrial energetics via the activation of purine salvage pathway. Our findings suggest that cellular bioenergetics are important in regulating NLRP3 activation, and XOR inhibition may be clinically relevant in NLRP3-related inflammatory diseases.

## Introduction

The NLRP3 inflammasome is a key complex to process and secrete IL-1β in response to a large variety of stimuli. These stimuli, damage-associated and pathogen-associated molecular patterns (DAMPs and PAMPs), include particulate factors such as monosodium urate (MSU) crystals, basic calcium phosphate crystals, cholesterol crystals, and soluble factors such as nigericin and extracellular ATP^1^. Several reports have demonstrated that the NLRP3-IL-1β pathway is strongly associated with many diseases such as gouty attack, atherosclerosis and diabetic nephropathy^2–4^. Thus, the NLRP3-IL-1β pathway may be a potential target for the treatment of the related-inflammatory diseases.

Mitochondria generate ATP and act as signalling hubs for apoptosis, ROS production and calcium mobilization. Upon NLRP3 activation, mitochondria also play critical roles^5^. Mitochondrial damage results in decrease in intracellular NAD^+^, a cofactor for NAD^+^-dependent sirtuin 2, resulting in the accumulation of acetylated α-tubulin. Acetylated α-tubulin mediates the transport of mitochondria and subsequent colocalization of ASC on mitochondria to NLRP3 on the endoplasmic reticulum, leading to the activation of NLRP3 inflammasome^6^. In addition, mitochondrial ROS (mitoROS) activates MAVS on the outer membrane, which associates with NLRP3 and promotes its oligomerization^7^. Furthermore, mitochondrial membrane depolarization causes the translocation of cardiolipin from the inner membrane to the outer membrane. The recruitment and interaction of NLRP3 with cardiolipin is essential for NLRP3 activation^8^. Mitochondrial DNA, mitochondria fission and fusion are also involved in NLRP3 activation^9,10^.

Xanthine oxidoreductase (XOR) is a rate limiting enzyme of purine metabolism, and catalyses the conversion of hypoxanthine/xanthine into xanthine/uric acid, respectively^11^. To date, we found that XOR mediates IL-1β secretion via mitoROS production, and thus febuxostat, a potent and highly selective inhibitor of XOR, inhibits IL-1β secretion by attenuating mitoROS production^12^. However, it is unclear whether febuxostat can inhibit mitoROS-independent IL-1β secretion.

In this study, we found that febuxostat inhibits mitoROS-independent as well as mitoROS-dependent IL-1β secretion. XOR inhibition by febuxostat improved cellular bioenergetics (restoring intracellular ATP (iATP) levels and ameliorating mitochondrial function), leading ultimately to the inhibition of IL-1β secretion and cell death in activated macrophages. These effects were mediated by the activation of salvage pathway. These findings suggest the importance of cellular bioenergetics in regulating NLRP3 activation, and the promising option of XOR inhibitor for treating NLRP3-related inflammatory diseases.

## Materials and Methods

### Preparation of bone marrow-derived macrophages (BMDMs)

Bone marrow cells were isolated from the tibia and femurs of C57BL/6 mice. For differentiation into BMDMs, the isolated cells were incubated for 6–7 days on Petri dishes in Dulbecco’s Modified Eagle Medium (DMEM, Thermo Fisher Scientific Inc., Waltham, MA) with 30% L929 conditioned medium, 10% FBS (PAA laboratories GmbH, Austria), 1% HEPES (Thermo Fisher Scientific Inc.) and 1% penicillin-streptomycin (Thermo Fisher Scientific Inc.). After differentiation, cells were detached using cold PBS, and plated for stimulation experiments in RPMI1640 medium (Thermo Fisher Scientific Inc.) with 10% FBS, 1% HEPES and 1% penicillin-streptomycin. Animal experiments were performed in strict accordance to the Swiss Federal Regulations or the Guiding Principles for the Care and Use of Laboratory Animals (Teijin Pharma Ltd.). The protocol was approved by the “Service de la consommation et des affaires vétérinaires du Canton de Vaud”, Switzerland, or by the Committee for Animal Experiments of the Teijin Institute for Biomedical Research.

### Preparation of human primary macrophages

Human primary macrophages were obtained according to the method described in Supplementary Information. All experimental procedures were conducted in accordance with the Guiding Principles for the Care and Use of Human Tissues (Teijin Pharma Ltd., Tokyo, Japan), and each experimental protocol was approved by the Committee for the Experiments with Human Tissues in the Teijin Institute for Bio-medical Research. All donors gave informed consent before collecting peripheral blood.

### Cell stimulation

BMDMs were primed overnight with 100 ng/mL of Pam3CSK4 (InvivoGen, San Diego, CA). The primed cells were incubated 30 min with vehicle, febuxostat (200 μM, Teijin Pharma Ltd., Tokyo, Japan) or MitoTEMPO (500 μM, ENZO Life Sciences, New York, NY) in incomplete medium (without FBS), and then stimulated with nigericin (2.5 μM, AppliChem GmbH, Germany or Sigma-Aldrich) or MSU crystal (0.25 mg/mL) for the indicated time. For intracellular ATP depletion, cells were treated with vehicle or febxostat in glucose-free RPMI1640 medium (Thermo Fisher Scientific Inc.) with 1% HEPES, 1% penicillin-streptomycin and 10 mM 2-deoxyglucose (2DG; Sigma, St. Louis, MO).

### Immunoblot

Equal amounts of supernatants or cell lysates were loaded to SDS-PAGE, and immunoblot was performed by anti-cleaved IL-1β (Asp117) Ab (Cell Signaling Technology Inc., Danvers, MA), anti-Caspase-1 (p20) Ab (Casper-1, AdipoGen, San Diego, CA) or anti-NLRP3 Ab (D4D8T, Cell Signaling Technology Inc.).

### ELISA

For detection of IL-1β level in supernatant, mouse IL-1β ELISA kit (eBioscience, Inc., San Diego, CA) was used according to the manufacturer’s instructions.

### Measurement of mitochondrial ROS level

Mitochondrial ROS level was measured with dihydrorhodamine 123 (DHR123, Thermo Fisher Scientific Inc.), which is a nonfluorescent ROS indicator that can passively diffuse across membranes where it is oxidized in the mitochondria to fluorescent rhodamine 123. Briefly, BMDMs in half area 96-well clear bottom black plate were primed, pretreated 30 min with vehicle, febuxostat or MitoTEMPO, and then stimulated 90 min with medium, nigericin or MSU. After stimulation, cells were loaded 30 min with 2 μM DHR123, and fluorescence intensity on 500 nm (excitation) and 536 nm (emission) was measured with fluorescence plate reader.

### Measurement of LDH

LDH in supernatant was measured using CytoTox-ONE™ Homogeneous Membrane Integrity Assay (Promega) according to the manufacturer’s instructions. LDH release (%) was calculated by using the following formula. LDH release (%) = [(value in sample) − (background)]/[(value in Triton X-100-treated sample) − (background)] × 100.

### Measurement of cell viability

Cell viability was measured with double staining kit (Dojindo, Kumamoto, Japan) based on calcein-AM staining for live cells and propidium iodide (PI) staining for dead cells. Briefly, BMDMs plated in half area 96-well clear bottom black plate were prime, pretreated 30 min with vehicle, febuxostat or MitoTEMPO, and then stimulated 6 h with medium or nigericin. For dead cell preparation for positive control, cells were treated 1 min with ethanol (70%). After stimulation, cells were stained 15 min with 2 μM calcein-AM and 4 μM PI, and fluorescence intensities on 485 nm (excitation)/535 nm (emission) for calcein-AM and 530 nm (excitation)/620 nm (emission) for PI were measured with fluorescence plate reader.

### Measurement of intracellular adenylates

Intracellular ATP was measured using CellTiter-Glo™ Luminescent Cell Viability Assay (Promega, Madison, WI) according to the manufacturer’s instructions. Luminescence was measured with a luminometer and ATP concentration was calculated by using ATP standard curve. Intracellular ATP content was expressed as nmol/mg protein by protein concentration that determined by BCA method. For HPLC analysis, intracellular metabolites were extracted with 10% perchloric acid and neutralized with 3 M potassium carbonate. Extracted metabolites were analysed using YMC-Triart C18 column.

### Measurement of mitochondrial membrane potential

Mitochondrial membrane potential was measured with tetramethylrhodamine, ethyl ester (TMRE, Life Technologies), which is a fluorescent dye that is readily sequestered by active mitochondria depending on mitochondrial membrane potential. Depolarized or inactive mitochondria have decreased membrane potential and then fail to sequester TMRE. Briefly, BMDMs in 96-well half area clear bottom black plate were pretreated 30 min with vehicle, febuxostat or MitoTEMPO, and then stimulated 90 min with medium or nigericin. After stimulation, cells were loaded 30 min with 500 nM TMRE, and fluorescence intensity on 549 nm (excitation) and 575 nm (emission) was measured with fluorescence plate reader.

### Measurement of intracellular uric acid

Amplex® red uric acid/uricase assay kit (Thermo Fisher Scientific Inc.) was used to detect intracellular uric acid level according to the manufacturer’s instructions. Intracellular uric acid level was expressed as nmol/mg protein by protein concentration that determined by BCA method.

### Metabolome analysis

Primed BMDMs were pretreated 30 min with vehicle or febuxostat, and then stimulated 90 min with medium or nigericin. Cells were washed with 5% mannitol and the metabolites were extracted with methanol containing internal standard. The metabolites were analysed by CE-TOFMS (Agilent CE-TOFMS system) and CE-QqQMS (Agilent CE system and Agilent 6460 TripleQuad LC/MS). Absolute value of metabolite was determined, and the relative ratios of nigericin-treated group to medium group, and of febuxostat/nigericin-treated group to nigericin-treated group were calculated to be expressed as the heat map.

### Measurement of mitochondrial respiration capacity

Primed BMDMs plated in Seahorse XFp Cell Culture Miniplate (Agilent Technologies, Santa Clara, CA) were equilibrated in XF Base Medium Minimal DMEM (Agilent Technologies) supplemented with 1 mM pyruvate (Thermo Fisher Scientific Inc.), 25 mM glucose (Sigma) and 2 mM glutamine (Thermo Fisher Scientific Inc.). Then, cells were treated for 30 min with vehicle or febuxostat (200 μM), and stimulated for 90 min with nigericin (2.5 μM). Oxygen consumption rate (OCR) was measured with the Seahorse XFp Extracellular Flux analyzer (Agilent Technologies) by injecting 1 μM oligomycin (Sigma), 1 μM FCCP (abcam, Cambridge, UK), and 0.5 μM antimycin A (abcam)/0.5 μM rotenone (LKT Laboratories, Inc., St. Paul, MN). OCR was expressed as picomoles per minute.

### Statistical analysis

All data are expressed as mean ± SD. For two-group comparisons, student’s *t* test was used. For multiple comparisons, one-way ANOVA followed by Tukey’s test were used to compare between each groups. All data were statistically analyzed using GraphPad PRISM software version 6.01 (GraphPad, La Jolla, CA). Differences with a probability value of <0.05 were considered significant.

## Results

### Febuxostat inhibits IL-1β secretion by mitochondrial ROS-independent and -dependent mechanisms

**Figure 1:**
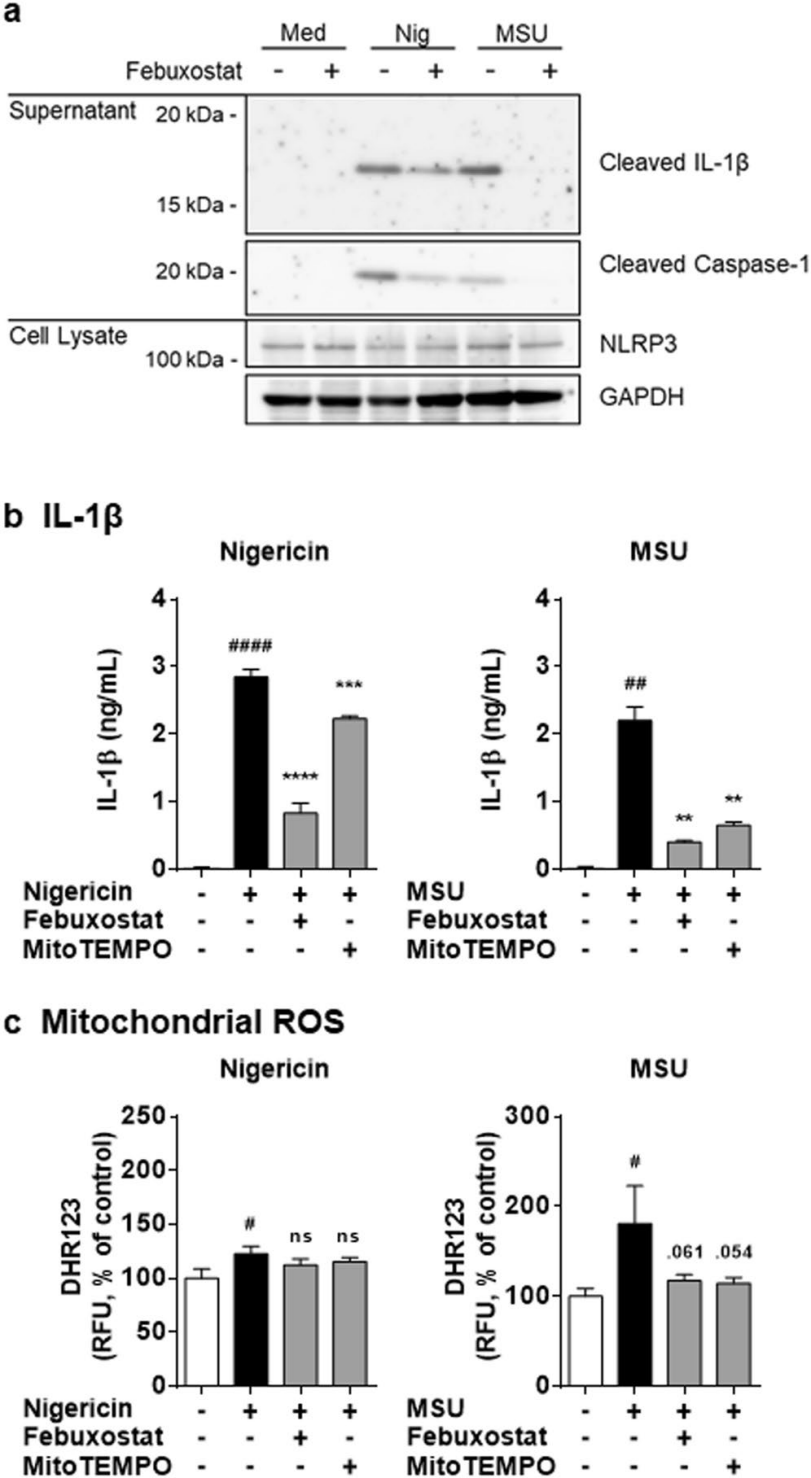
Febuxostat inhibits IL-1β secretion in mitochondrial ROS-independent and -dependent manners. (**a**) Primed BMDMs were pretreated 30 min with vehicle or febuxostat, and then stimulated 2 h with nigericin or MSU. Supernatant and cell lysate were used for immunoblotting. Data are representative of two independent experiments in which the same data were obtained. (**b**) Primed BMDMs were pretreated 30 min with vehicle, febuxostat or MitoTEMPO, and then stimulated 2 h with nigericin or MSU. IL-1β in the supernatant was analysed by ELISA. (**c**) Primed BMDMs were pretreated 30 min with vehicle, febuxostat or MitoTEMPO, and then stimulated 90 min with nigericin or MSU. After stimulation, cells were loaded with DHR123. Data are representative of three independent experiments performed in triplicate and shown as mean ± SD. ^#^*p* < 0.05, ^##^*p* < 0.01, ^####^*p* < 0.0001 versus non-stimulated control. **p < 0.01, ***p < 0.001, ****p < 0.0001 versus nigericin or MSU-treated group. ns, not significant.

We have previously demonstrated that macrophage secretion of IL-1β upon NLRP3 inflammasome activation involved mitoROS production by XOR. Accordingly, we found that pharmacological inhibition of XOR by febuxostat decreased mitoROS and IL-1β secretion^12^. Consistent with our previous study, febuxostat effectively inhibited IL-1β secretion by nigericin or MSU, depending on NLRP3, caspase-1 and ASC (Fig. 1a, and see Supplementary Fig. S1). The inhibitory effects of febuxostat on IL-1β secretion were also confirmed in human primary macrophages (see Supplementary Fig. S2). We provide now additional data showing that febuxostat also acts on mitoROS-independent mechanisms of IL-1β secretion. Nigericin-induced IL-1β secretion was largely independent of mitoROS production as the mitochondrial scavenger MitoTEMPO only had a minor inhibitory effect on nigericin-induced IL-1β secretion (Fig. 1b). On the other hand, MitoTEMPO significantly inhibited MSU-induced IL-1β secretion (Fig. 1b). Differential contribution of mitoROS in nigericin- and MSU-mediated cellular responses was also supported by the fact that nigericin was a poor inducer of mitoROS formation whereas MSU was a much stronger inducer (Fig. 1c). MitoROS production by MSU was inhibited by both febuxostat and MitoTEMPO (Fig. 1c), whereas as expected in nigericin-treated cells, febuxostat and MitoTEMPO did not change mitoROS levels. Altogether, these results suggested that upon nigericin treatment, febuxostat inhibits IL-1β secretion via a mechanism that is distinct from mitoROS suppression.

### Febuxostat inhibits cell death in mitochondrial ROS-independent manner

**Figure 2:**
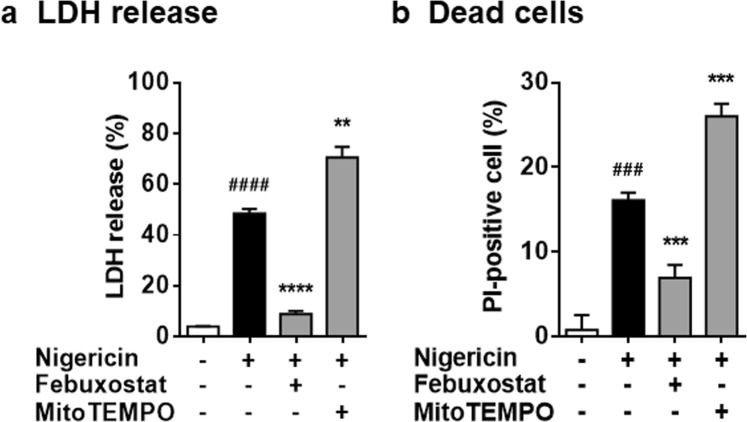
Febuxostat inhibits cell death in mitochondrial ROS-independent manner. (**a**) Primed BMDMs were pretreated 30 min with vehicle, febuxostat or MitoTEMPO, and then stimulated 2 h with nigericin. LDH activity in the supernatant was measured. (**b**) Primed BMDMs were pretreated 30 min with vehicle, febuxostat or MitoTEMPO, and then stimulated 6 h with nigericin. After stimulation, cells were stained with calcein for living cell and PI for dead cell. Data are representative of three independent experiments performed in triplicate and shown as mean ± SD. ^###^*p* < 0.001, ^####^*p* < 0.0001 versus non-stimulated control. **p < 0.01, ***p < 0.001, ****p < 0.0001 versus nigericin-treated group.

We next examined the effects on cell death, because NLRP3 activators have been reported to disturb membrane integrity and induce cell death^13^. As shown in Fig. 2a, treatment for 2 h with nigericin led to significant increase in LDH release, indicating the disturbance of cell membrane integrity. In addition, longer treatment (6 h) caused cell death (Fig. 2b). Febuxostat significantly suppressed nigericin-induced LDH release and cell death, whereas MitoTEMPO did not (Fig. 2a,b). These results demonstrated that nigericin induces cell death, as well as IL-1β secretion, independently of mitoROS production, and that febuxostat protects cells from cell injury and death upon nigericin treatment.

### Febuxostat restored intracellular ATP levels and inhibited IL-1β secretion on NLRP3 inflammasome activation

**Figure 3:**
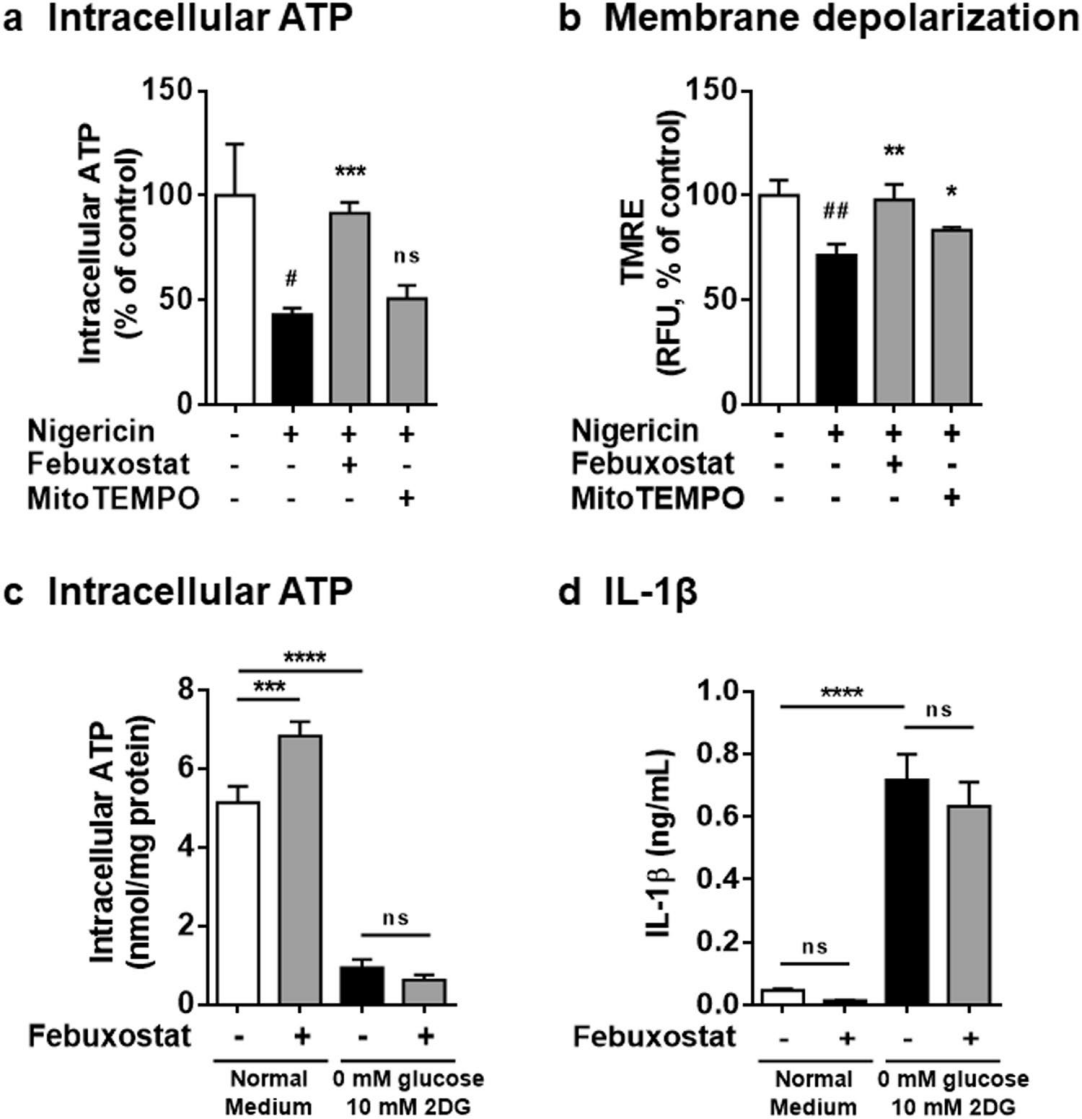
Restored intracellular ATP is involved in the inhibitory effects of febuxostat. (**a**) Primed BMDMs were pretreated 30 min with vehicle, febuxostat or MitoTEMPO, and then stimulated 2 h with nigericin. Intracellular ATP was measured by luminescence method. (**b**) Primed BMDMs were pretreated 30 min with vehicle, febuxostat or MitoTEMPO, and then stimulated 90 min with nigericin. After stimulation, cells were loaded with TMRE. Data are representative of three independent experiments performed in triplicate and shown as mean ± SD. ^#^*p* < 0.05, ^##^*p* < 0.01 versus non-stimulated control. *p < 0.05, **p < 0.01, ***p < 0.001 versus nigericin-treated group. ns, not significant. (**c**) Primed BMDMs were incubated 6 h with glucose-free medium containing 10 mM 2DG in the presence of vehicle or febuxostat. RPMI1640 medium containing 10 mM glucose was used as normal medium. Intracellular ATP was measured. (**d**) IL-1β in the supernatant was analysed by ELISA. Data are representative of two independent experiments performed in triplicate and shown as mean ± SD. ***p < 0.001, ****p < 0.0001. ns, not significant.

We have previously shown that nigericin induces iATP loss, mitochondrial membrane potential (Δψm) depolarization, finally leading to IL-1β secretion, which is independent of mitROS production^14^. Thus, we examined the effects of febuxostat on iATP content and Δψm. Nigericin treatment caused approximately 60% decrease in iATP compared to non-treated control. Febuxostat prevented the lowering of iATP levels on nigericin treatment whereas MitoTEMPO failed to do so (Fig. 3a). Similarly, febuxostat significantly protected nigericin-induced depolarization of Δψm and this effect was greater than that of MitoTEMPO (Fig. 3b). As shown in Fig. 1a,b, febuxostat inhibited nigericin-induced IL-1β secretion in the same condition. Considering that iATP depletion induces Δψm depolarization, finally leading to IL-1β secretion in primed BMDMs^14^, these data suggested that restored iATP by febuxostat leads to the protection of Δψm depolarization and IL-1β secretion. We next examined whether febuxostat inhibits IL-1β secretion in artificial iATP depleting condition that febuxostat failed to increase iATP level (Fig. 3c). In this condition, there was no effect of febuxostat on IL-1β secretion (Fig. 3d), implying that the importance of restored iATP by febuxostat on IL-1β inhibition. Taken together, these data suggested that restored iATP by febuxostat contributes to Δψm maintenance and IL-1β inhibition. However, further studies are needed to clarify the detailed mechanisms.

### Febuxostat restored intracellular ATP by activating the purine salvage pathway

**Figure 4:**
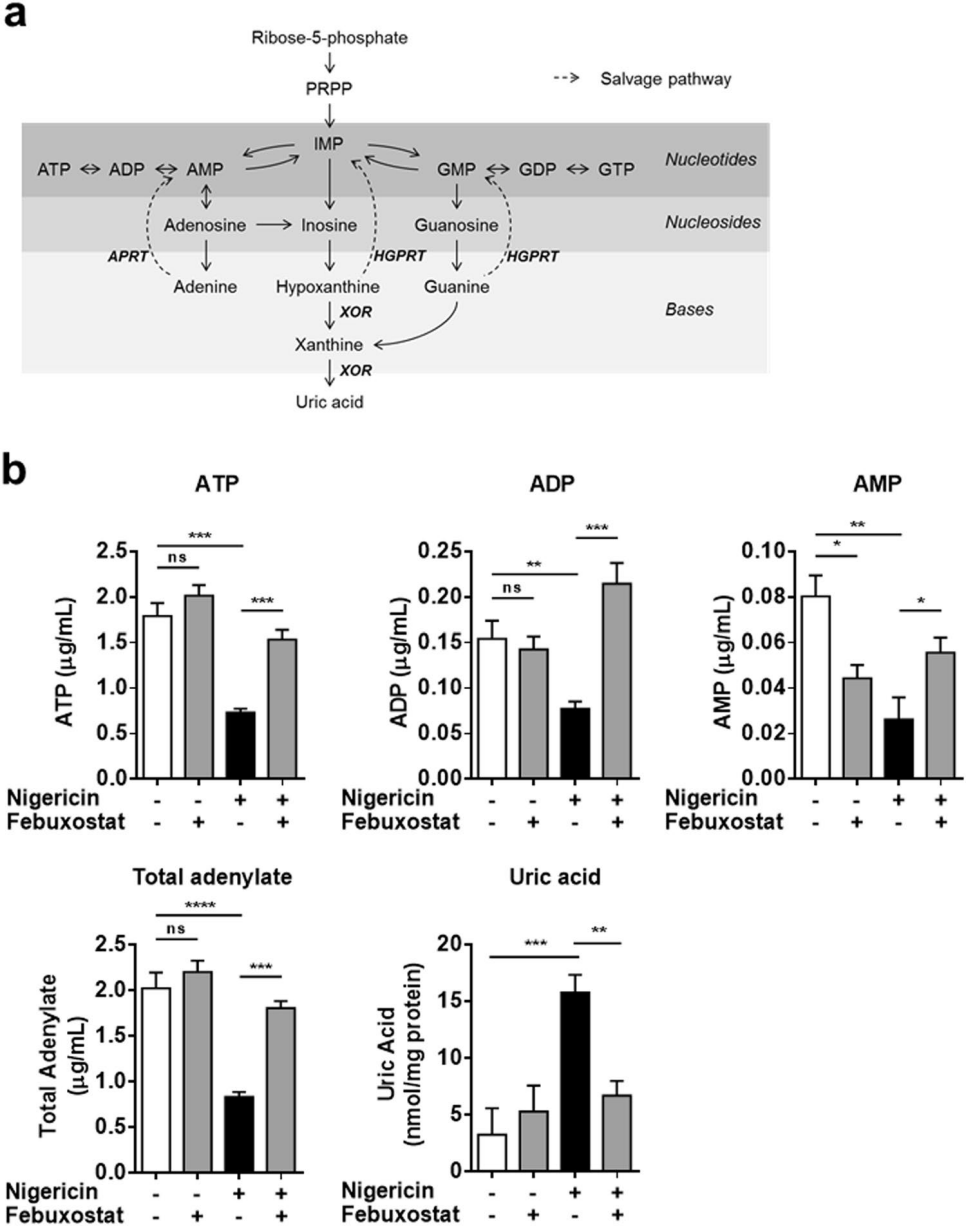
Febuxostat restored intracellular ATP by activating salvage pathway. (**a**) Schema of purine metabolism. (**b**) Primed BMDMs were pretreated 30 min with vehicle or febuxostat, and then stimulated 90 min with nigericin. Intracellular ATP, ADP and AMP were measured by HPLC method. Total adenylate was calculated. Intracellular uric acid level was measured. Data are representative of two independent experiments in which the same data were obtained and shown as mean ± SD. *p < 0.05, **p < 0.01,***p < 0.001, ****p < 0.0001. ns, not significant.

To determine the mechanism underlying the restoration of iATP by febuxostat, we investigated the intracellular levels of purine metabolites. XOR acts at the final steps of the purine metabolism pathway. XOR inhibition leads to the accumulation of hypoxanthine, in turn resulting in the activation of the salvage pathway, a recycling pathway of purine bases to form the purine nucleotides (Fig. 4a). Nigericin treatment led to the decreases in ATP, ADP, AMP and total adenylate (ATP + ADP + AMP), and an increase in uric acid level, indicating that nigericin induced purine catabolism. Febuxostat significantly inhibited the rise in uric acid, and restored ATP, ADP, AMP and total adenylate cell content (Fig. 4b). In addition to the adenylate, GTP was also restored by febuxostat (see Supplementary Fig. S3). These data demonstrated that febuxostat restores iATP by activating the purine salvage pathway.

### Febuxostat improves cellular bioenergetics

**Figure 5:**
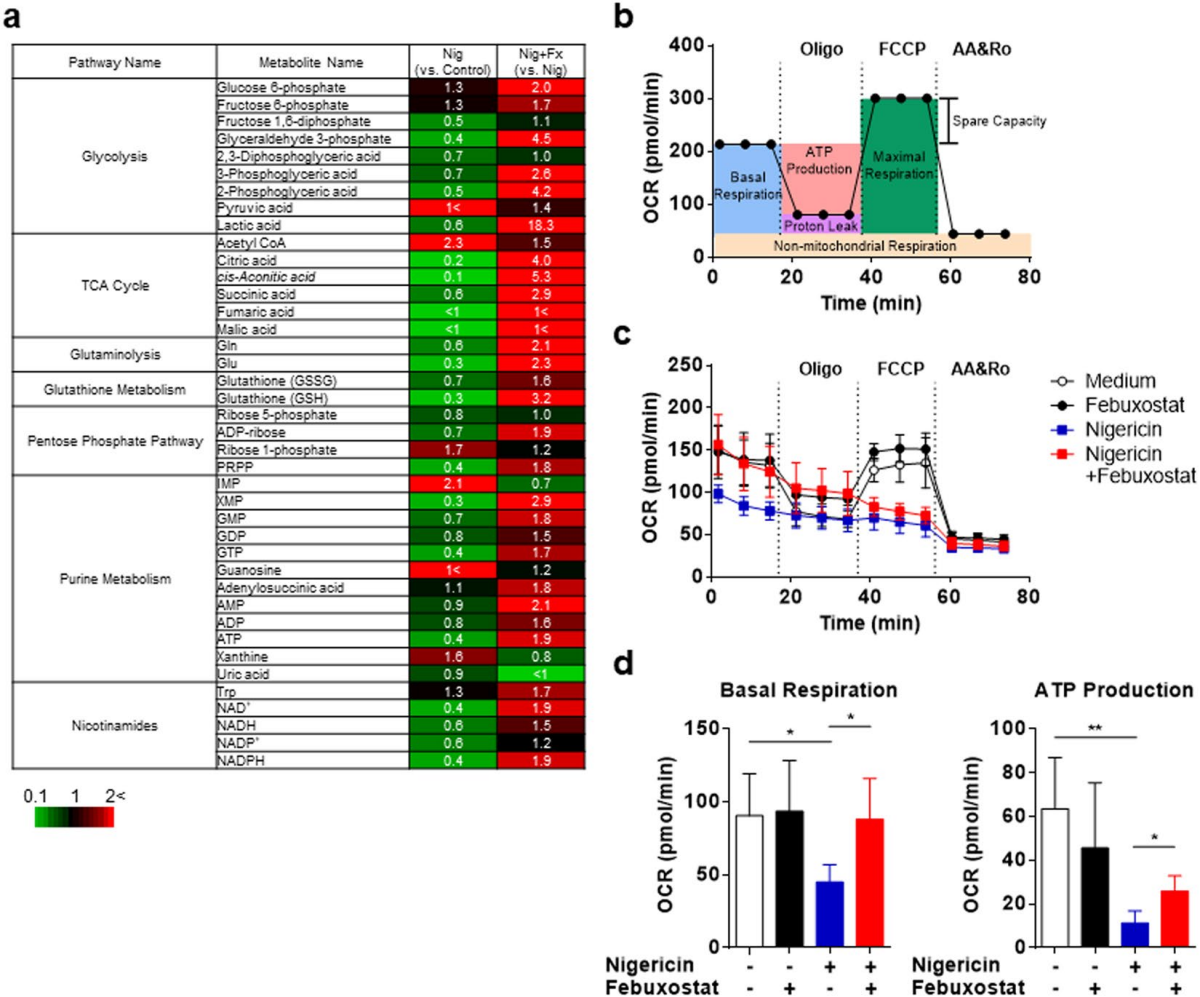
Febuxostat improves cellular bioenergetics. (**a**) Heatmap image of metabolome data. Primed BMDMs were pretreated 30 min with vehicle or febuxostat, and then stimulated 90 min with nigericin. Cell extracts were used for metabolome analysis. Red indicates increased expression whereas green indicates decreased expression. (**b**) Illustration of mitochondrial respiratory capacity. (**c,d**) Primed BMDMs were pretreated 30 min with vehicle, febuxostat or MitoTEMPO, and then stimulated 90 min with nigericin. OCR was measured with the Seahorse XFp Extracellular Flux analyser. Basal respiration and ATP production were calculated by subtracting OCR value at 73.7 and at 34.3 min from OCR value at 14.6 min in (**c**), respectively. Data are shown as mean ± SD of four independent experiments in singlet. *p < 0.05, **p < 0.01.

We found that XOR inhibition by febuxostat affects purine metabolites, leading to change in adenylates (ATP, ADP, AMP), crucially involved in various cellular events. Thus, we hypothesized that febuxostat could extensively affect intracellular metabolites upon NLRP3 inflammasome activation. To examine this, metabolome analysis was performed using stimulated BMDMs treated 90 min with nigericin and/or febuxostat. Metabolome analysis revealed that nigericin perturbed various intracellular pathways: glycolysis, TCA cycle, glutaminolysis, pentose phosphate pathway, purine metabolism, nicotinamides, and glutathione metabolism (Fig. 5a). Pyruvic acid and Acetyl CoA were increased by nigericin, suggesting that TCA cycle was stalled. Although uric acid levels were not increased in our metabolome analysis, upstream metabolites of uric acid, IMP and xanthine, were increased, suggesting that nigericin stimulates the purine degradation. Finally we could confirm by metabolome analysis that nigericin also induced down-regulation of adenylates (as found in Fig. 4b). Most importantly, febuxostat treatment improved almost all of nigericin-induced changes of intracellular metabolites (Fig. 5a). These effects of febuxostat may be due to improved cellular energetics such as restored iATP. Intermediate metabolites of TCA cycle, which are utilized for electron transport chain (ETC) followed by oxidative phosphorylation (OxPhos) in mitochondria, were increased by febuxostat (Fig. 5a). Finally, we sought to determine whether febuxostat improves mitochondrial function. To this end we examined the mitochondrial respiratory capacity by measuring the oxygen consumption rate (OCR). Figure 5b illustrates the mitochondrial respiratory capacity followed by oligomycin (Oligo, ATP synthase inhibitor), FCCP (uncoupler) and antimycin/rotenone (AA/Ro, complex III/I inhibitor, respectively). Nigericin functions as K^+^/H^+^ exchanger in mitochondria, thus leading to the disturbance of ETC. In nigericin-treated cells, basal respiration, ATP production, and maximal respiration were depleted compared with non-treated control cells (Fig. 5c). Maximal respiration was not changed by febuxostat, suggesting that febuxostat is not a direct antagonist of nigericin. Febuxostat significantly restored the basal respiration and ATP production in nigericin-treated cells (Fig. 5d). Collectively, these results demonstrated that febuxostat improves mitochondrial function and cellular bioenergetics.

## Discussion

NLRP3 inflammasome activation leads to IL-1β secretion by multiple mechanisms, one of which is on mitoROS production^15,16^. We have previously shown that XOR inhibition attenuated IL-1β release by reducing ROS generation^12^. Here we show that febuxostat, a potent XOR inhibitor, reduced IL-1β secretion by both mitoROS-dependent and independent pathways. Febuxostat restored iATP levels and improved mitochondrial dysfunction in nigericin-treated cells. Cellular bioenergetics were also improved.

Changes of cellular bioenergetics have been reported to be involved in IL-1β secretion during NLRP3 activation^17–19^. In this study, febuxostat improved nigericin-induced disturbance of cellular bioenergetics. This was supported by the findings that iATP was restored by febuxostat and that the metabolites of glycolysis and TCA cycle was increased by febuxostat (Figs. 3a, 4b and 5a). Metabolomic analysis using plasma from XOR knockout mice revealed that intermediates of TCA cycle such as pyruvate, citrate, aconirate, α-ketoglutarate, fumarate and malate were up-regulated compared to wild-type mice^20^. In addition, febuxostat up-regulates the expression of glutamate oxaloacetate transaminase 2, which converts glutamate to α-ketoglutarate, in spinal cords from experimental autoimmune encephalomyelitis mice^21^. Thus, XOR inhibition improved cellular bioenergetics.

The purine salvage pathway plays an important role in ATP maintenance, as evidenced by the low levels of ATP in Lesch-Nyhan syndrome patients who have a mutation of hypoxanthine-guanine phosphoribosyltransferase (HGPRT), a crucial enzyme in the salvage pathway^22^. XOR inhibition results in the accumulation of hypoxanthine, which in turn leads to the activation of salvage pathway via HGPRT^23,24^. Given these previous reports, the restoration of iATP by febuxostat is mainly due to the activation of salvage pathway because febuxostat increased not only ATP but also GTP (see Supplementary Fig. S3). In addition, OxPhos in mitochondria may also contribute the restoration of iATP because Oligo-sensitive ATP production was increased by febuxostat (Fig. 5d). ATP synthase phosphorylates ADP resulting in ATP production. Thus, increased ADP supply by XOR inhibition may cause ATP production in mitochondria.

The mechanism involved in the induction of the purine salvage pathway by febuxostat is linked to its high specificity for XOR. Febuxostat is a non-purine XOR inhibitor which does not affect HGPRT activity^25^. The accumulation of hypoxanthine by febuxostat induces the production of IMP via HGPRT activity. We also studied the effect of a purine analogue XOR inhibitor, allopurinol. Allopurinol, a hypoxanthine isomer, by its chemical structure could also act as a competitive inhibitor of HGPRT^26,27^. Indeed, in macrophages allopurinol did not restore decreased intracellular levels of ATP induced by nigericin, strongly suggesting that the purine salvage pathway is inhibited by allopurinol (see Supplementary Fig. S4).

Mitochondria dysfunction is major event mediating NLRP3 activation and subsequent IL-1β secretion^15,16^. It has been reported that mitochondrial damage leads to a decrease in intracellular NAD^+^, which in turn results in the inactivation of NAD^+^-dependent sirtuin 2, the accumulation of acetylated α-tubulin, the transport of mitochondria, and finally the activation of NLRP3^6^. Consistent with this report, intracellular NAD^+^ was significantly decreased by nigercin. Interestingly, febuxostat significantly restored NAD^+^ level. In addition, NAD^+^ replenishment inhibited nigericin-induced IL-1β secretion and restored iATP levels (see Supplementary Fig. S5). These results suggest that the restoration of iATP and NAD^+^ by XOR inhibition is likely to modulate the various events in mitochondria upon NLRP3 activation. However, further studies are needed to understand the detailed mechanisms.

**Figure 6:**
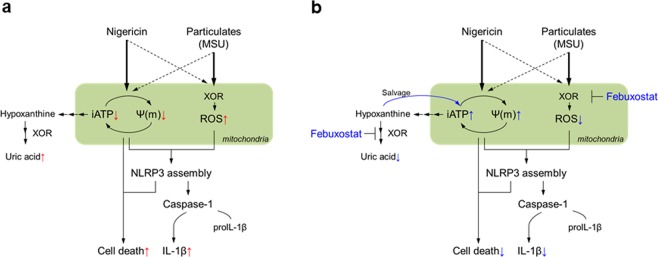
Schema of the mechanisms underlying the inhibitory effect of febuxostat on NLRP3 inflammasome activation. (**a**) MSU mainly activates mitoROS-dependent pathway, while nigericin mainly activates an iATP loss-dependent pathway (Black arrows in bold). In particular, nigericin induces mitochondrial dysfunction, and iATP loss by the disturbance of oxidative phosphorylation, finally leading to IL-1β secretion and cell death. Concomitantly, iATP degrades into uric acid via purine degradation pathway. Red arrows indicate the effects of NLRP3 inflammasome activators. (**b**) In the case of stimuli with mitoROS production, such as MSU crystals, febuxostat inhibits IL-1β secretion through the inhibition of mitoROS production. In the case of nigericin stimulation, febuxostat inhibits IL-1β secretion by restoring iATP and improving mitochondrial function via the salvage pathway activation. Blue arrows indicate the effects of febuxostat on NLRP3 inflammasome activation pathways.

In conclusion, we have showed that XOR inhibition by febuxostat attenuates IL-1β secretion in activated macrophages (Fig. 6). In the case of stimuli with mitoROS production, such as MSU crystals, the inhibitory effect on IL-1β is mainly mediated by inhibition of mitoROS production. In stimuli which do not induce mitoROS production, such as nigericin, restored iATP and improved mitochondrial function play important roles. Our findings suggest the importance of cellular bioenergetics in regulating NLRP3 activation, and the promising option of XOR inhibition in controlling inflammatory diseases.

## Supplementary information

Supplementary information
