# Supplementary material for: Febuxostat, a Xanthine Oxidoreductase Inhibitor, Decreases NLRP3-dependent Inflammation in Macrophages by Activating the Purine Salvage Pathway and Restoring Cellular Bioenergetics
Source: Sci Rep. 2019 Nov 21;9:17314. doi: 10.1038/s41598-019-53965-x (PMC6872548; doi:10.1038/s41598-019-53965-x)
Supplement: Supplementary file 1 — Supplementary information [file 41598_2019_53965_MOESM1_ESM.pdf]

Febuxostat, a Xanthine Oxidoreductase Inhibitor, Decreases NLRP3-dependent Inflammation in Macrophages by Activating the Purine Salvage Pathway and Restoring Cellular Bioenergetics

Johji Nomura*, Tsunefumi Kobayashi*, Alexander So†, Nathalie Busso†

*Pharmacology Research Department, Teijin Institute for Bio-medical Research, Teijin Pharma Limited, Hino, Tokyo, Japan, †Service of Rheumatology, Department of Musculoskeletal Medicine, Centre Hospitalier Universitaire Vaudois, University of Lausanne, Lausanne, Switzerland

*Corresponding author: Johji Nomura, tel; +81-42-586-8190, fax; +81-42-587-5517, j.nomura@teijin.co.jp

Supplementary Information

Materials and Methods

THP-1 cell experiment

THP1 cells stably expressing short hairpin (sh) RNA against lamin (Mock), NLRP3 (shNLRP3), caspase-1 (shCasp1), and ASC (shASC) were used as described before[1]. The resulting knocked down THP-1 cells were primed 72 h with 200 nM PMA, and incubated 30 min with vehicle or febuxostat, and 2 h stimulated with nigericin or MSU.

Preparation of human macrophages

Human monocytes were purified from peripheral blood from healthy donors with the Ficoll-Paque gradient and subsequent MACS Monocyte Isolation Kit (Miltenyi Biotec, Bergisch Gladbach, Germany). Human macrophages were obtained by culturing monocytes for 7 days in the presence of 50 ng/mL human M-CSF (Miltenyi Biotec). All experimental procedures were conducted in accordance with the Guiding Principles for the Care and Use of Human Tissues (Teijin Pharma Ltd., Tokyo, Japan), and each experimental protocol was approved by the Committee for the Experiments with Human Tissues in the Teijin Institute for Bio-medical Research. All donors gave informed consent before collecting peripheral blood. Human macrophages primed overnight with 200 ng/mL of ultrapure LPS (Invivogen) were incubated 30 min with vehicle or 200 μM febuxostat, and then stimulated with nigericin (1.25 μM,

AppliChem GmbH, Germany) and MSU crystal (0.25 mg/mL) for the indicated time.

HPLC analysis

For HPLC analysis, intracellular metabolites were extracted with 10% perchloric acid and neutralized with 3 M potassium carbonate. Extracted metabolites were analysed using YMC-Triart C18 column.

Supplementary Fig. S1

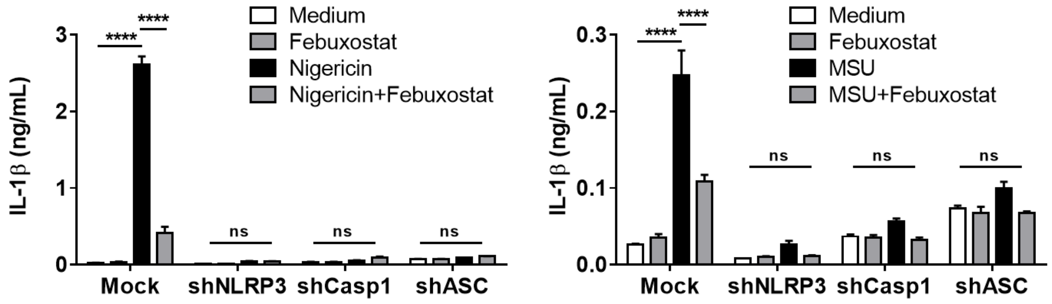

Supplementary Figure S1. Febuxostat inhibits NLRP3 inflammasome. Primed THP-1 cells (Mock, shNLRP3, shCasp1 or shASC) were pretreated 30 min with vehicle or febuxostat, and then stimulated 2 h with nigericin or MSU. IL-1β in the supernatant was analysed by ELISA. Data are representative of two independent experiments performed in triplicate and shown as mean±SD. ****p<0.0001. ns, not significant.

Supplementary Fig. S2

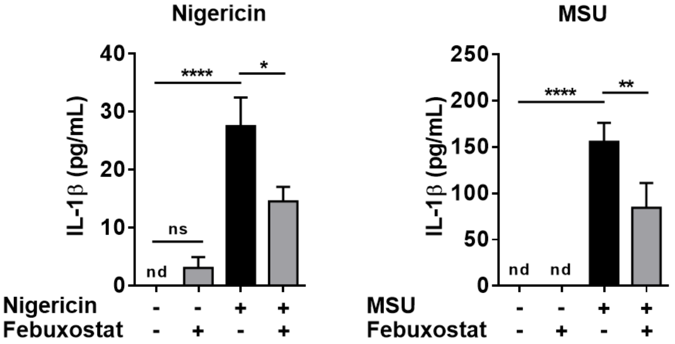

Supplementary Figure S2. Febuxostat inhibits IL-1β secretion in human primary macrophages. Primed human primary macrophages were pretreated 30 min with vehicle or febuxostat (200 μM), and then stimulated 2 h or 6 h with nigericin (1.25 μM) or MSU (0.25 mg/mL), respectively. IL-1β in the supernatant was analysed by ELISA, and the values were calculated as the average of triplicate. Data are shown as mean±SD of values obtained by using human macrophages from five (nigericin) or three (MSU) donors. **p*<0.05, ***p*<0.01, *****p*<0.0001. nd, not detected. ns, not significant.

Supplementary Fig. S3

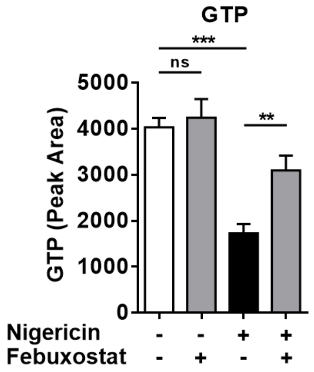

Supplementary Figure S3. Febuxostat restores intracellular ATP by activating salvage pathway. Primed BMDMs were pretreated 30 min with vehicle or febuxostat (200 μM), and

then stimulated 90 min with nigericin (2.5 μM). Intracellular GTP was measured by HPLC method. Data are representative of two independent experiments in which the same data were obtained and shown as mean±SD. ***p*<0.01,****p*<0.001. ns, not significant.

Supplementary Fig. S4

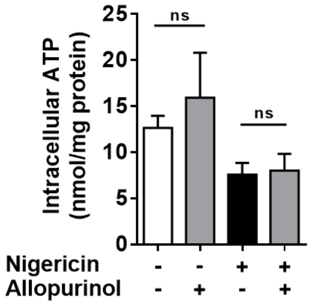

Supplementary Figure S4. Allopurinol fails to restore intracellular ATP. Primed BMDMs were pretreated 30 min with vehicle or allopurinol (1 mM), and then stimulated 90 min with nigericin (2.5 μM). Intracellular ATP was measured by luminescence method. Data are representative of two independent experiments in which the same data were obtained and shown as mean±SD. ns, not significant.

Supplementary Fig. S5

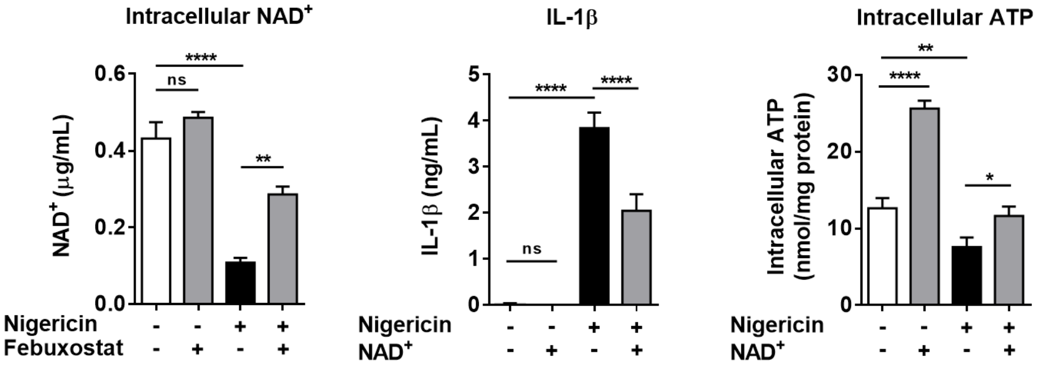

Supplementary Figure S5. Febuxostat restored NAD^+ content and replenished NAD^+ inhibited IL-1β secretion. Primed BMDMs were pretreated 30 min with vehicle, febuxostat (200 μM) or NAD^+ (20 mM), and then stimulated 90 min with nigericin (2.5 μM). Intracellular NAD^+ content was measured by HPLC method. IL-1β in the supernatant was analysed by ELISA. Intracellular ATP was analysed by luminescence method. Data are representative of two independent experiments in which the same data were obtained and shown as mean±SD. **p*<0.05, ***p*<0.01, *****p*<0.0001. ns, not significant.

Supplementary Figure S6

a. IL-1β

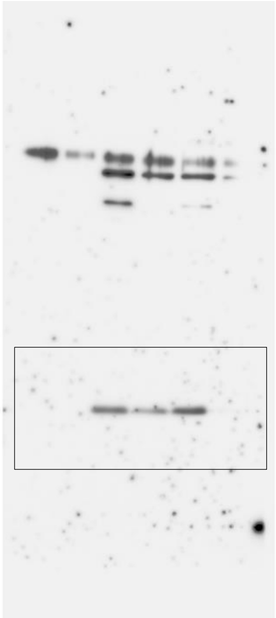

b. Caspase-1

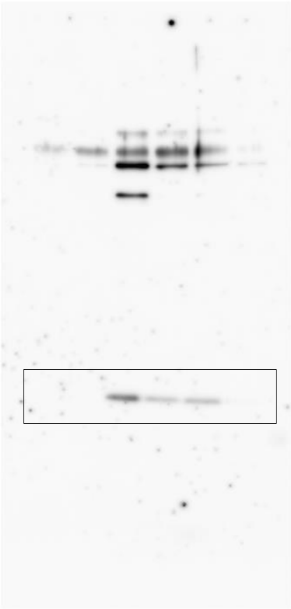

c. NLRP3

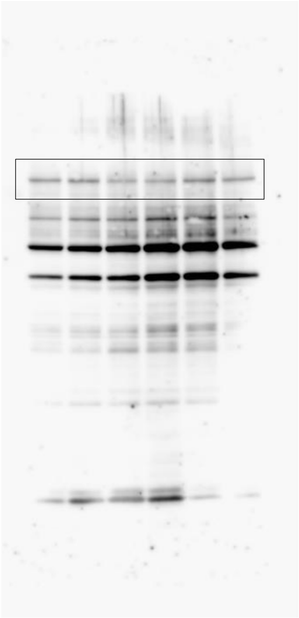

d. GAPDH

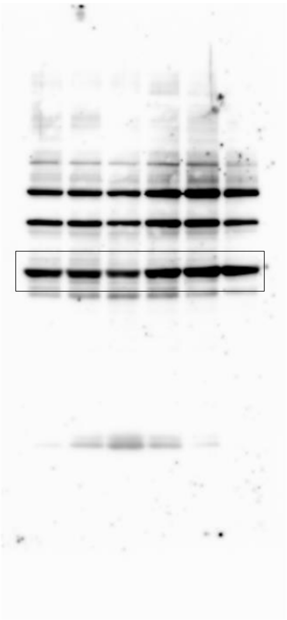

Supplementary Figure S6. Whole images of each blot.

Reference

1 Nomura, J., So, A., Tamura, M. & Busso, N. Intracellular ATP Decrease Mediates NLRP3 Inflammasome Activation upon Nigericin and Crystal Stimulation. *Journal of immunology* **195**, 5718-5724, doi:10.4049/jimmunol.1402512 (2015).
